# Wireless thin film transistor based on micro magnetic induction coupling antenna

**DOI:** 10.1038/srep18621

**Published:** 2015-12-22

**Authors:** Byoung Ok Jun, Gwang Jun Lee, Jong Gu Kang, Seunguk Kim, Ji-Woong Choi, Seung Nam Cha, Jung Inn Sohn, Jae Eun Jang

**Affiliations:** 1 Daegu Gyeongbuk Institute of Science and Technology (DGIST), Department of Information and Communication Engineering, Daegu, 711-873, Korea; 2 Advanced Naval Technology Center, Agency for Defense Development, Changwon, 645-600, Korea; 3 University of Oxford, Department of Electrical Engineering Science, Oxford, OXI 3PJ, U. K

## Abstract

A wireless thin film transistor (TFT) structure in which a source/drain or a gate is connected directly to a micro antenna to receive or transmit signals or power can be an important building block, acting as an electrical switch, a rectifier or an amplifier, for various electronics as well as microelectronics, since it allows simple connection with other devices, unlike conventional wire connections. An amorphous indium gallium zinc oxide (α-IGZO) TFT with magnetic antenna structure was fabricated and studied for this purpose. To enhance the induction coupling efficiency while maintaining the same small antenna size, a magnetic core structure consisting of Ni and nanowires was formed under the antenna. With the micro-antenna connected to a source/drain or a gate of the TFT, working electrical signals were well controlled. The results demonstrated the device as an alternative solution to existing wire connections which cause a number of problems in various fields such as flexible/wearable devices, body implanted devices, micro/nano robots, and sensors for the ‘internet of things’ (IoT).

As landline phones have been replaced to mobile phones, wireless has become one of the hot technical concepts recently, since it can provide both simplicity and convenience. It will inspire new device concepts, and it is essential to some applications. For example, a wire connection between a device implanted in a body and a control or a power supply system located externally can be a source of infection and an inconvenience for patients, which can be avoided with wireless functions^1^,^2^,^3^,^4^. Likewise, in flexible display systems, which are considered to be the most important hardware component for next generation smart phone systems, damage to the scan and data lines connecting the thin film transistors (TFTs) is one of the significant obstacles to their practical realization. The lines, in rows or columns, are easily broken by bending or folding, since the electrode shape is narrow (~μm level) and long (cm ~ m level). Although many approaches have been widely studied, most results do not provide an ultimate solution^5^,^6^,^7^,^8^,^9^. Alternatively, if the signal or the power is supplied using wireless technology, those issues could be easily solved. Wireless technology, enabled by a micro/nano-antenna, is already essential to micro/nano robots, because the scale of the micro/nano robot makes a wire connection impossible, due to the size of the wire^10^,^11^. Additionally, various wireless sensors are currently being intensively developed for IoT^12^,^13^,^14^,^15^,^16^, and much smaller wireless components also need to be developed for those applications. Therefore, a wireless TFT structure would be an important component, since it could be applied as an important building block in various devices, as an electrical switch, a rectifier or an amplifier, for various electronics with wireless functionality.

In this study, we investigated an amorphous indium gallium zinc oxide TFT (α-IGZO TFT) with a magnetic induction antenna structure. To incorporate a micro antenna structure into a TFT, a magnetic core (MC) of nickel (Ni) coated zinc oxide nano wires (ZnO NWs) was added to the micro antenna structure. This enhanced the power or the delivered signal efficiency, which was reduced as antenna size becomes smaller. The electrical characteristics and the α-IGZO TFT structure were optimized to improve the wireless transferred power efficiency, considering the increase of self-resonance frequency produced by the antenna size effect. The suggested wireless TFT structure can be a core technology in the field of flexible devices, implantable systems and micro robots, etc.

## Results

### Characterization of the micro antenna with various MC structures

**Figure 1:**
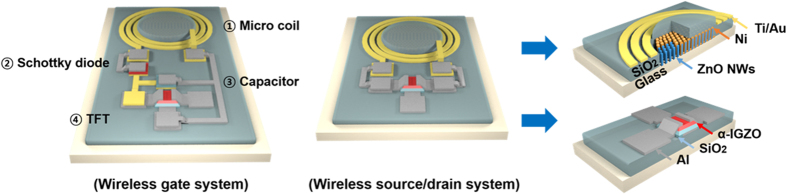
Schematic diagram of wireless thin film transistor concept.

The schematic diagrams of the wireless α-IGZO TFT are shown in Fig. 1. The main body consists of a micro-antenna and TFT. The antenna structure that receives or transmits electrical signals or power outside the system is connected directly to a source/drain or gate electrode of the TFT.

For wireless signal processing or sensing, the antenna is connected to source/drain electrodes. Signals transmitted to the antenna from the outside can be modified or filtered by the TFT. Small detected signals, such as a biological signal from the brain, can also be amplified and then transmitted to the outside, as well. If the antenna is connected to the gate of the TFT, electrical on-off states can be controlled by wireless signals, so that it can be employed as a wireless electrical switch for various applications such as a flexible display or wearable device. To realize various functions, a diode and a capacitor can be added to the body structure of the wireless TFT, depending on the application and experimental set-up. A smaller antenna size is appropriate for many applications. For a wireless TFT structure in a flexible display system, a micro-level antenna size is required, considering the pixel size and bending radius. One side effect of this micro size antenna is the decrease of transmission efficiency that occurs with scaling down the size of antenna. To improve this low efficiency, a magnetic core (MC) structure was added to the micro antenna design to increase the magnetic flux density without changing the size of the antenna structure.

**Figure 2:**
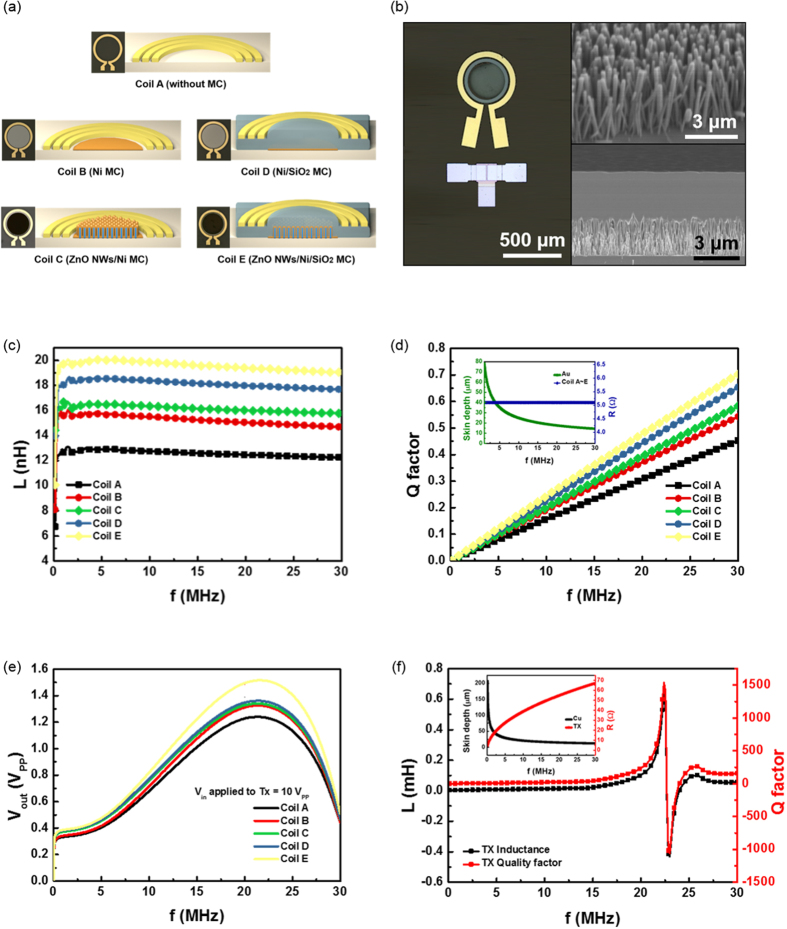
(**a**) Schematic diagram of the wireless α-IGZO TFT concept. (**b**) Optical microscope (OM) images of the fabricated wireless α-IGZO TFT (left) and scanning electron microscope (SEM) image of ZnO nanowire and core structure (right). (**c**) Inductances of various micro coils according to MC effects. (**d**) Quality factors of micro coils according to MC effects. (**e**) Wirelessly transferred output voltage of various micro coil designs according to the frequency using solenoid type TX antenna. (**f**) Inductance and quality factor of TX.

Figure 2(a) shows various designs of the MC structure. Ni was chosen as the magnetic material. Ferrites or other materials have a higher magnetic permeability, however the permeability of ferrites decreases dramatically in the high frequency region due to Snoek’s limit^17^,^18^. Metallic magnetic materials are good candidates to compensate the limit. For some designs, ZnO NWs have been used for the base structure under the Ni film since the nanowires can easily increase the effective area, which leads to a high surface to volume ratio, even when the same thickness of metal is employed in the antenna structure. The MC structure was formed on the same plane as the antenna or under the antenna structure (Fig. 2(a)). Photo images of the antenna structures are shown in Fig. 2(b). After growing the vertically aligned ZnO NWs on the center area of the antenna structure, Ni film was deposited on the ZnO NWs. To confirm the electromagnetic properties of the various MC structures, their inductances were measured using a network analyzer (Agilent technology E5061B) connected to a test fixture (Agilent technology 16047E) in the frequency range from 5 Hz to 30 MHz. The presence of a high-permeability magnetic material in the center of the coil increases the inductance by providing a low-reluctance path for the magnetic flux lines, which reduces the loss of magnetic fields escaping from the center^19^. Figure 2(c) shows the measured inductances of various antenna designs. The results show that the inductances of all of the micro coils with various MC structures are much larger than the inductance of the simple micro coil design without MC. In the MC structures, increasing the effective area density of Ni with ZnO NWs and insulating the MCs with SiO_2_ led to effectively increasing the inductances from ~15 nH (coil B) to ~16 nH (coil C) and ~18 nH (coil D), respectively. Even when the area density of coil C was larger than coil D, the higher inductance was observed at coil C. While metallic magnetic materials are good candidates for compensating Snoek’s limit, permeability is also decreased at high frequency due to eddy current losses caused by their high conductivity. Hence, SiO_2_ was deposited on the MC structures to suppress the eddy current effect by insulating them with dielectric oxides^20^. It can be confirmed that insulating the MCs is an important factor for increasing magnetic properties by restraining the eddy current effect. With coil E, the highest inductance of ~20 nH was observed due to both effects, a value almost 2 times higher than the inductance of the coil without MC. The wireless power and signal transmission (WPST) efficiency can be expressed by


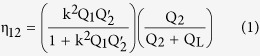


where k, Q_1_, Q_2_ and Q_L_ are the coils coupling coefficient, the quality factors of transmitting antenna (TX) in the primary part, receiving antenna (RX) and load in the secondary part, respectively. The quality factor in the secondary part can be driven as 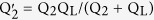
21. In the equation of WPST efficiency, we can see that an increase of Q_2_ can improve the WPST efficiency. The AC resistances of the micro coils were extracted by the equation 
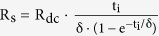
 where R_dc_ is the DC resistance, t_i_ is the conductor thickness and δ is the skin depth^22^. To obtain the quality factors of the micro coil designs according to the MC effects, the DC resistances were measured. The measured DC resistances of all the coil designs were almost the same since they all shared basically the same coil features, and the calculated AC resistance remained constant (5–5.2 Ω) without any changes from DC resistance, as the frequency increased up to 30 MHz. AC resistance mainly depends on the skin effect according to the frequency, but the skin effect did not affect the AC resistances of our micro coil designs. This is because the calculated skin depth of gold (Au) 
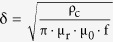
 declines by about 14 μm at 30 MHz, which is thicker than the thickness of the micro coil, where ρ_c_ is the resistivity of the conductive material, μ_r_ is the relative permeability of the conductor, μ_0_ is the permeability of space and f is the frequency^23^. Hence the quality factor follows the tendency of inductance. As shown in Fig. 2(d), the quality factors of all the micro coil designs increase with a similar tendency approaching the self-resonance frequency generally observed at GHz level, due to their tiny size. Among the designs, the coil E structure has the highest quality factor, as expected. Figure 2(e) shows the wirelessly transferred output voltage of various micro coil designs with varying the frequency of TX from 1 KHz to 30 MHz when applying the input voltage of 10 V_PP_ (peak-to-peak voltage) to the TX. A solenoid type TX with a centimeter level size wound by 1 mm thick copper (Cu) wires was used to reduce the error from misalignment between TX and RX during the measurement of WPST efficiency. Coil E shows the highest transferred voltage, ~1.5 V_PP_, which result can be predicted easily from the inductance and the Q factor value. All case show the highest transferred voltage around 22.4 MHz. It is mainly due to the resonance frequency of TX antenna as shown in Fig. 2(f).

### Characterization of α-IGZO TFT

Considering the increase in self-resonance frequency with decreasing antenna size, it is necessary to drive the TFT at high frequency, and hence, we chose an amorphous indium gallium zinc oxide TFT (α-IGZO TFT) structure, which shows higher mobility than an α-Si TFT^24^.

**Figure 3:**
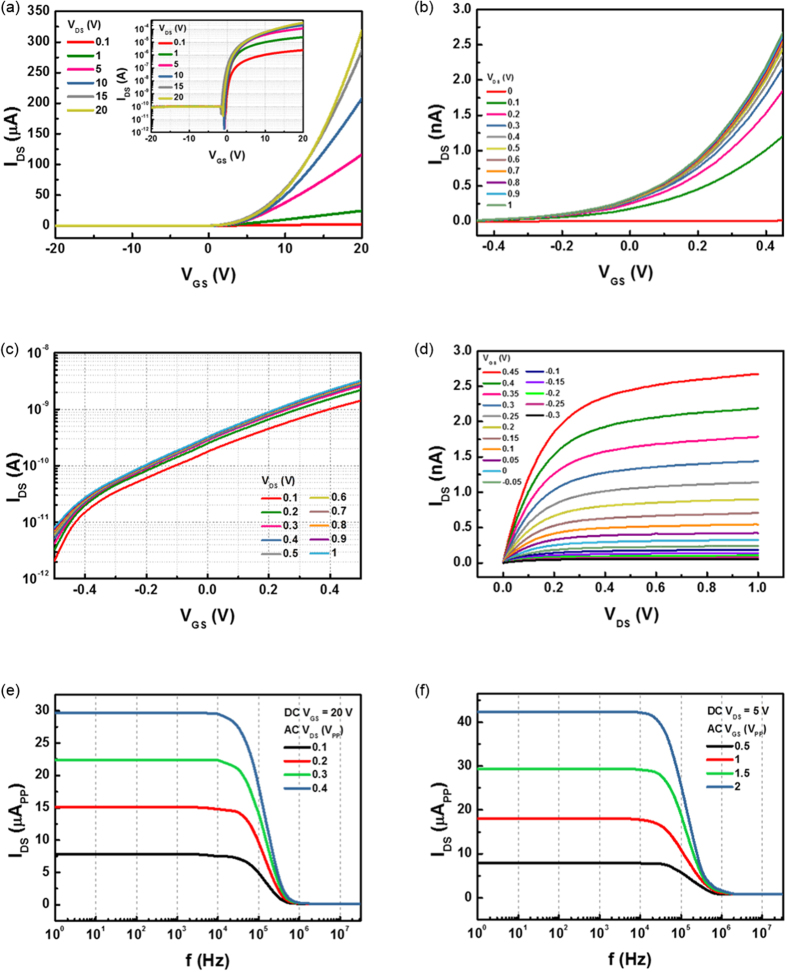
Electrical characteristics of the α-IGZO TFT. W/L is 10 (100 μm/10 μm) (**a**) Transfer characteristic of α-IGZO TFT. (**b**) Linear scale transfer curve of α-IGZO TFT in mV levels of V_DS_ and V_GS_. (**c**) Log scale transfer curve of α-IGZO TFT in mV levels of V_DS_ and V_GS_. (**d**) Output curve of the α-IGZO TFT in mV levels of V_DS_ and V_GS_. (**e**) Frequency response characteristic at the source/drain (S/D) of the α-IGZO TFT. (**f**) Frequency response characteristic at the gate of the α-IGZO TFT.

The electrical performances of the fabricated α-IGZO TFT were characterized as shown in Fig. 3. Figure 3(a) shows the transfer characteristic of the α-IGZO TFT with optimized structure for this wireless concept. The corresponding field-effect mobility μ_FE(sat)_ was obtained with 13 cm^2^/V_s_ calculated as W_c_(100 μm), L_c_(10 μm), and C_i_(17.4 nF) which are the channel width, length, and gate capacitance per unit area, respectively. The following on-off ratio was about ~10^7^. This is comparable to some other reported performance^25^,^26^. In our wireless TFT concept, a threshold voltage (V_th_) close to 0 V is required for wireless switching because the transmitted output voltage from TX is in the ~mV level. The V_th_ can be controlled by the thickness of the TFT channel, t_c_. The V_th_ was optimized as the t_c_ changed from 5 nm to 40 nm (Fig. S2). The V_th_ closes to 0 V at 40 nm channel thickness. Then, the electrical characteristics were reconfirmed in mV level of source/drain voltage (V_DS_) and gate voltage (V_GS_), which are the transferred output voltage levels to micro coil designs in Fig. 3(b–d). At the mV levels, the corresponding on-off ratio was about ~10^3^, and however, it is adequate for switching in our concept.

To verify the electrical characteristics of α-IGZO TFT in the various frequency regions, the drain current (I_DS_) was measured in response to the frequency when applying AC voltage to the source/drain and the gate, respectively, as shown in Fig. 3(e,f). In the both cases, the I_DS_ gradually decreases over the 10 kHz up to MHz level. Generally the cutoff frequency of α-IGZO TFT exhibits in the range between kHz and MHz^27^. Hence, the proper operating frequency limit of α-IGZO TFT can be around 10 ~ 100 kHz for the wireless system. The higher cutoff frequency are attributed to the high mobility in the short channel length. The short channel effect is shown in Fig. S2. The result proves the improved frequency response characteristic of α-IGZO TFT by decreasing channel length, which better performance is observed at α-IGZO TFT with channel length of 10 μm.

### Performance of wireless α-IGZO TFT with different antenna designs

**Figure 4:**
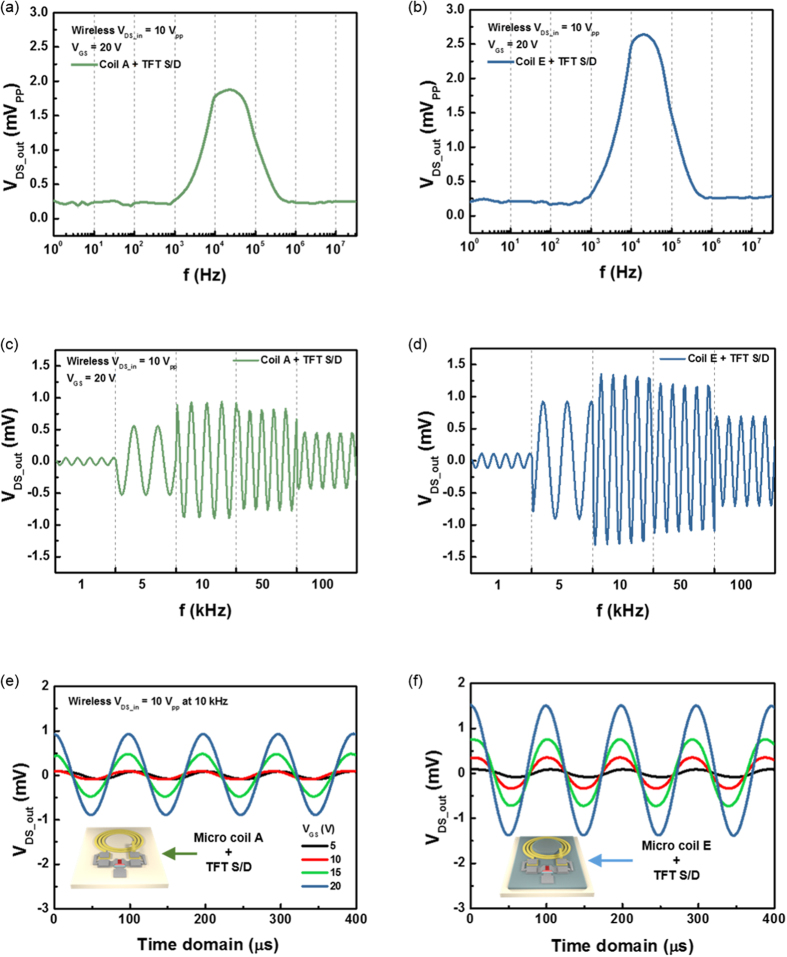
Electrical characteristics of the wireless S/D of the α-IGZO TFT. (**a**) V_DS_ with various driving frequency characteristic of micro coil A connected with S/D of the α-IGZO TFT. (**b**) V_DS_ with various driving frequency characteristic of micro coil E connected with S/D of the α-IGZO TFT. (**c**) AC signal induced in drain electrode by coil A from 1 kHz to 100 kHz (**d**) AC signal induced in drain electrode by coil E from 1 kHz to 100 kHz. (**e**) AC Transfer characteristic of micro coil A connected with S/D of the α-IGZO TFT. (**f**) AC Transfer characteristic of micro coil E connected with S/D of the α-IGZO TFT.

To confirm the antenna performance of the TFT structure, the antenna structure and the source/drain electrodes of the α-IGZO were connected, and then, the V_DS_ was measured when the input voltage was supplied to the TX with 10 V_PP_. Figure 4(a,b) represents electrical characteristics of junction between source and drain for transferred AC signal with various frequency from 1 Hz to 30 MHz and Fig. 4(c,d) shows much clearer waveform of the wirelessly transferred signals in operable frequency region from 1 kHz to 100 kHz using antenna designs A and E, respectively. The proper operating frequency with the highest output V_DS_ was observed at around 20 ~ 30 kHz in both cases. As expected from the result of Fig. 2, the coil E design shows much better signal transfer characteristics than coil A for the TFT connection due to the MC effect. The WPST efficiency of the antenna can be maximized at the resonance frequency. Therefore, as the working frequency is increased up to the resonance frequency, the WPST efficiency is enhanced. The resonance frequency of our micro size antenna and TX solenoid antenna are about 1 ~ 3 GHz depending on designs and 22.4 MHz, respectively. However, the performance of the α-IGZO TFT is degraded with increasing working frequency over 10 KHz as shown inn Fig. 3(e). Therefore, these two different relations for the working frequency suggests the optimized working frequency level. The gating effect for wireless transmissive signals to source/drain is shown in Fig. 4(e,f). V_GS_ was changed from 5 V to 20 V when applying the 10 V_PP_ at 10 kHz to TX. With the increasing gate bias, the conductive channel is opened widely, so that the current flow formed by the wireless transmission is increased in both antenna designs. From the result, the wirelessly received V_DS_ was well controlled by V_GS_, and output V_DS_ was increased up to about 2.6 mV_PP_ at 20 V of V_GS_, as shown in Fig. 4(f).

### Electrical characteristics of α-IGZO TFT employing a wireless gate structure

**Figure 5:**
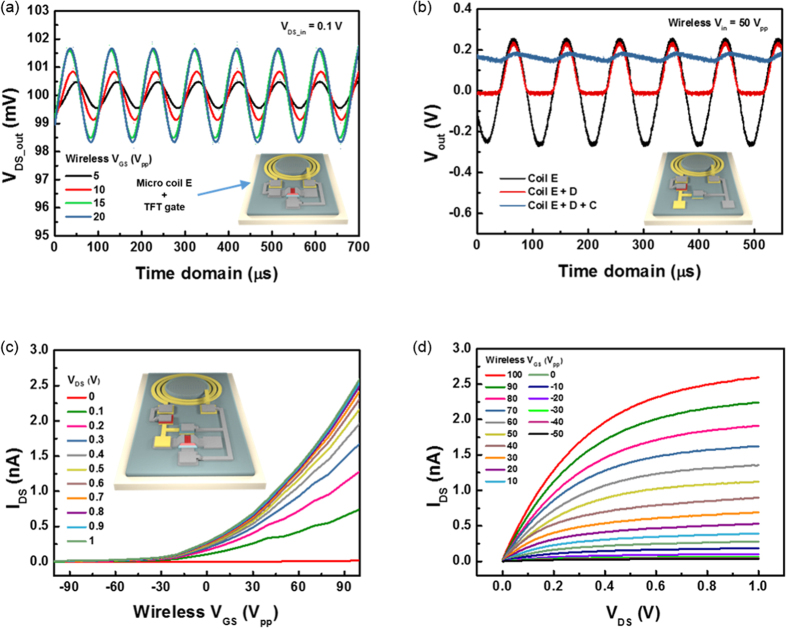
Characteristics of the wireless gate of the α-IGZO TFT. (**a**) AC transfer characteristics of the micro coil (coil E) connected with the gate of the α-IGZO TFT. (**b**) Electrical characteristics of the wireless rectifying system. D and C mean a diode and a capacitor, respectively. (**c**) Transfer characteristics of the wireless rectifying system connected with the wireless gate of the α-IGZO TFT. (**d**) Output characteristic of the wireless rectifying system connected with the gate of the α-IGZO TFT.

The performance of the wireless gate was investigated via the electrodes of the coil E design. The gate electrodes of the α-IGZO TFT were connected to the antenna structure. The measured output V_DS_ is represented in Fig. 5(a). The applied voltage to TX antenna increased from 5 V_PP_ to 20 V_PP_ for TX. By increasing the applied bias to TX, the output voltage change of the source/drain is increased since the transmissive gate swing voltage induced by the antenna is increased. At 20 V_PP_ of TX antenna swing, the out voltage difference is about 3.4 mV. For wireless gate control, especially to make an electrical ‘on-off’ switch, the wirelessly delivered voltage must be rectified close to the DC bias. Hence, coil E was connected with a Schottky diode (D) in series for half-wave rectification, and a capacitor (C) with a capacitance of 1 μF was connected with them for a wave delay closer to DC.

The schematic diagram and the characteristics of the wireless rectifying system are represented in Fig. 5(b). Although a Schottky diode generally has a little higher leakage current at reverse bias than a p-n junction diode, it can have a higher cut-off frequency with a simple structure, so it is appropriate with a wireless α-IGZO TFT structure. The output voltage from the antenna E structure (RX) is about 0.5 V_PP_ at 50 V_PP_ supplied to TX. As expected from DC and AC electrical characteristics of the diode structure shown in S7 (supporting information), by the Schottky diode structure, the positive component of the output bias from the RX is remained, while most of negative parts are removed. The small negative signals come from the reverse leakage current of the diode structure. The sine wave form is changed finally to a dc-like result by the capacitor. The rectified voltage is about 0.2 V through the Schottky diode and capacitor. The transfer and output characteristics of the wireless α-IGZO TFT were measured with these rectifying components. In Fig. 5(c,d), the graphs correspond well with the traditional characteristics of a TFT. In comparison to the transfer and output characteristics of the α-IGZO TFT in Fig. 3(a,b), the results in Fig. 5(c,d) are reasonable. The measurement results indicate that the conductance of the TFT is well controlled by the wireless V_GS_. The characteristics of wireless α-IGZO TFT can be extracted by the change of V_GS_ received wirelessly in RX versus the change of generated I_DS_ in TFT. The corresponding on-off ratio is about ~10^3^, and this level can be enhanced by higher WPST efficiency and better RF matching skills. Therefore it can be applied as an electrical switch for wearable, implantable and flexible devices. The manufacturing process of the wireless system can be fully simplified since a Schottky diode with higher speed performance, and a capacitor, can be easily formed using α-IGZO and a SiO_2_ or Si_3_N_4_ insulator. Therefore, the procedure is available simultaneously during the fabrication of the α-IGZO TFT at low temperature. Moreover, the WPST efficiency was presented with relatively lower performance in this example, but it can be improved by optimizing the process for the TX and RX matching parameter, as mentioned before.

## Discussion

We chose near-field magnetic induction (MI) coupling as the wireless technique since it can easily transmit power or signal through water as well as ambient air^28^,^29^. If general RF technology is used for devices implanted in the body, the attenuation of signal is too severe due to the body’s water component. The human body is composed of about 70% water. Because of the relative permittivity difference between the air (ε_r_ ≈ 1) and the water (ε_r_ ≈ 80), the RF electromagnetic wave does not propagate the signal or the power well from the air to the human body and vice versa. Unlike the RF technique, a magnetic wave produced by a magnetic field (*H-field*) propagates into a channel for the MI power transmission. The intensity of the magnetic wave strongly depends on magnetic permeability (*μ*). Almost all media including air, water, wood and even metals such as gold (Au) and aluminum (Al) have a similar relative magnetic permeability (*μ*_*r*_ ≈ 1). Therefore, a signal or power transmission based on the MI technology will experience little loss during propagation between the air and the human body. Additionally, the inductive coupling method is appropriate to the wireless TFT concept given the wide driving frequency characteristics. If we choose magnetic resonance coupling in which two resonators are coupled electromagnetically, and the energy in one resonator is transmitted to the other through an evanescent mode wave, it can give much higher transmission efficiency than the inductive coupling mode. However, a number of technical challenges still exist before this technology can be transitioned to a successful commercial product, because the technology has high efficiency only when the source and receiving coils are aligned coaxially. Moreover, the resonance frequency of the fabricated micro antenna structure is about 1 ~ 5 GHz. That value is very harsh for a general TFT structure or even some transistors based on single crystal material.

We have studied the wireless TFT structure as an important electrical building block for various applications. The MC with a Ni structure supported by ZnO NWs formed under the plane of the micro coil could effectively improve the inductance value of the micro antenna structure, since it provides the MC structure with a higher effective area density with good insulating effect. The α-IGZO TFT design with high μ_FE(sat)_ was optimized for wireless driving, as well. The micro coil antenna connected with the source/drain of the α-IGZO TFT effectively delivered the signal to the source/drain and the signal was well controlled by the gate bias. The proper frequency was observed to be ~10 kHz due to the ‘trade-off’ relationship between the working frequency of the antenna and the TFT. For wireless gate control, especially to create on-off states, the wirelessly delivered voltage was rectified to be close to DC bias using a Schottky diode and capacitor, which can be produced simultaneously during the fabrication of the α-IGZO TFT at low temperature. The wireless rectifying system was adopted to the gate of the α-IGZO TFT and the wireless α-IGZO TFT was successfully operated as a micro wireless switching device with a corresponding on-off ratio of about ~10^3^. From the experimental results, we can confirm that our wireless TFT concept has high potential for applications in various fields such as flexible devices, implantable systems and micro robots.

## Methods

### Fabrication of the micro coils with various MC structures and α-IGZO TFT

The micro coil antenna was fabricated as a circular flat spiral type coil with about 50/500 nm thickness of titanium (Ti)/gold (Au), respectively, on glass substrate using a radio frequency (RF) magnetron sputtering system and photolithography process. The flat spiral coil offers more flexibility in defining characteristics on flexible substrates, such as polyimide^30^. By applying an optimization process for the RX and TX parameters, such as the number of turns, width, thickness of coil and spacing between coil traces, the wireless power and signal transmission (WPST) efficiency can be significantly enhanced^22^.

Then, various MC structures were formed in the center of the fabricated micro coil. The MC features are shown in Fig. 2(a). A nano-structure of ZnO NWs served as a frame to increase the area density of the Ni layer. The ZnO NWs were vertically grown with lengths of ~3 μm in nutrient solution, which was a mixture of hexamethylenetetramine and zinc nitrate hexahydrate^31^,^32^,^33^,^34^. After that, a Ni layer with a thickness of 100 nm was coated and patterned on the vertical ZnO NWs. To realize the α-IGZO TFT, firstly, 150 nm thick Al was patterned on a glass substrate using an RF magnetron sputtering system and a photolithography process for formation of the gate electrode. The gate insulator layer of SiO_2_ was deposited by plasma enhanced chemical vapor deposition (PECVD) with a thickness of 200 nm, and then, it was etched by using buffered oxide etchant (BOE) 6:1. Finally, the α-IGZO active layer with a thickness of 40 nm was patterned and Al source/drain electrodes were formed. Al material was used to form the Ohmic-contact at the metal-semiconductor junction because Al has a work function that is well matched with n-type α-IGZO material^35^. (More details are in Supporting information section: S3~S6).

### Data analysis of micro coils with various MC structures

The quality factors according to the MC effects were calculated as follow:


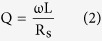


where ω is the angular frequency, L is the inductance and R_s_ is the AC resistance, which is extracted by the equation:


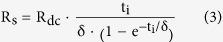


where R_dc_ is the DC resistance, t_i_ is the conductor thickness and δ is the skin depth, which is defined as:


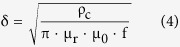


where ρ_c_ is the resistivity of the conductive material, μ_r_ is the relative permeability of the conductor, μ_0_ is the permeability of space and f is the frequency. The WPST efficiency, η_12_, can be expressed by the equation below:


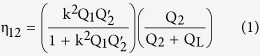


where k, Q_1_, Q_2_ and Q_L_ are the coils coupling coefficient, the quality factors of TX in primary part, RX and load in secondary part respectively. The quality factor in secondary part can be driven as:


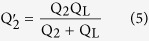


### Data analysis of α-IGZO TFT

A field-effect mobility, μ_FE(sat)_, was calculated by the following equation:


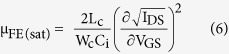


where, W_c_, L_c_, and C_i_ are the channel width, length, and gate capacitance per unit area.

## Additional Information

**How to cite this article**: Jun, B. O. *et al.* Wireless thin film transistor based on micro magnetic induction coupling antenna. *Sci. Rep.* **5**, 18621; doi: 10.1038/srep18621 (2015).

## Supplementary Material

Supplementary Information
