# Supplementary material for: Wireless thin film transistor based on micro magnetic induction coupling antenna
Source: Sci Rep. 2015 Dec 22;5:18621. doi: 10.1038/srep18621 (PMC4686891; doi:10.1038/srep18621)
Supplement: Supplementary Information [file srep18621-s1.pdf]

Supplementary Information

Wireless thin film transistor based on micro magnetic induction coupling antenna

Byoung Ok Jun[1], Gwang Jun Lee[1], Jong Gu Kang[1,2], Seung Uk Kim[1], Ji Woong Choi[1], Seung Nam Cha[3], Jung In Sohn[3], and Jae Eun Jang[1,*]

[1]Daegu Gyeongbuk Institute of Science and Technology (DGIST), Department of Information and Communication Engineering, Daegu, 711-873, Korea

[2]Advanced Naval Technology Center, Agency for Defense Development, Changwon, 645-600, Korea

[3]University of Oxford, Department of Electrical Engineering Science, Oxford, OXI 3PJ, U. K

*jang1@dgist.ac.kr

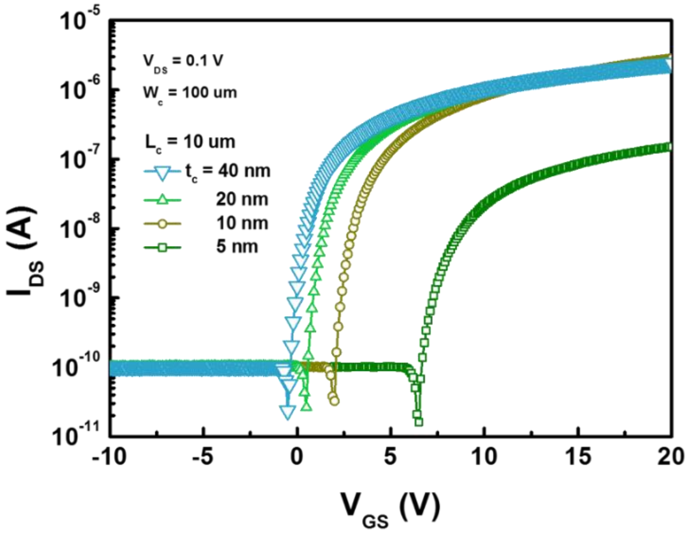

Supplementary Figure S1 | Outcurve of α-IGZO TFTs according to channel thickness for threshold voltage with close to 0 V. To achieve the threshold voltage (V_{th}) with close to 0 V, the α-IGZO TFTs with various thickness of channel (t_c) were fabricated and characterized by transfer curve to confirm the V_{th}. The V_{th} was verified as the t_c changed from 5 nm to 40 nm (channel width (W_c) =100 μm) when applying the gate voltage (V_{GS}) from -10 V to 20 V and the source/drain voltage (V_{DS}) of 0.1 V. The result shows that the V_{th} with close to 0 V can be achieved at 40 nm.

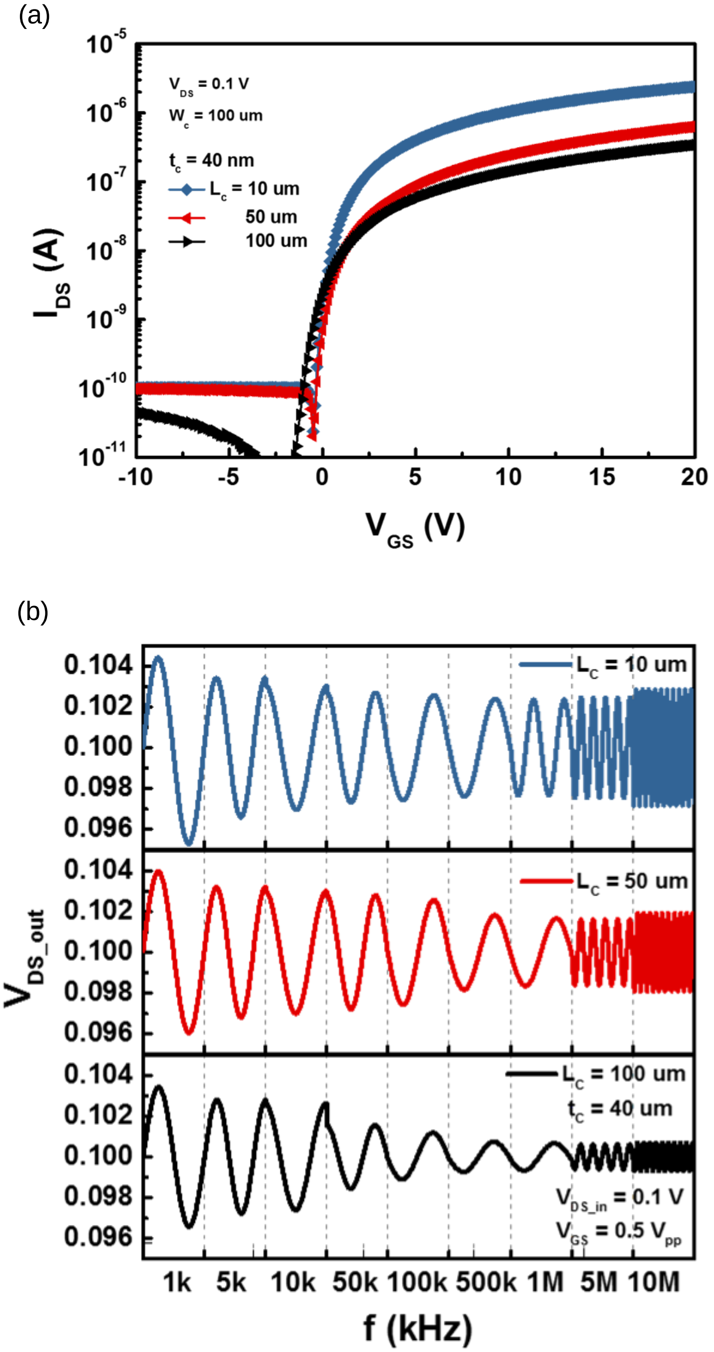

Supplementary Figure S2 | Frequency characteristics of α-IGZO TFTs according to channel length. The cutoff frequency of α-IGZO TFT is followed by $f_T \approx \frac{\mu_{FE}|V_{GS}-V_{th}|}{2\pi {L_c}^2}$, which mainly depends on field-effect mobility (μ_{FE}) and channel length (L_c). We already achieved the α-IGZO TFT with comparable μ_{FE} and the α-IGZO TFTs with various L_c were fabricated and characterized to obtain the better frequency performance of the α-IGZO TFT. The proper t_c was fixed at 40 nm and L_c changed from 10 um to 100 um (W_c=100 μm and V_{DS} = 0.1 V). As decreasing L_c, the resistance of channel became decreased as shown in Fig S2 (a) and the α-IGZO TFT with the better frequency performance could be obtained at L_c of 10 um as shown in Fig S2 (b).

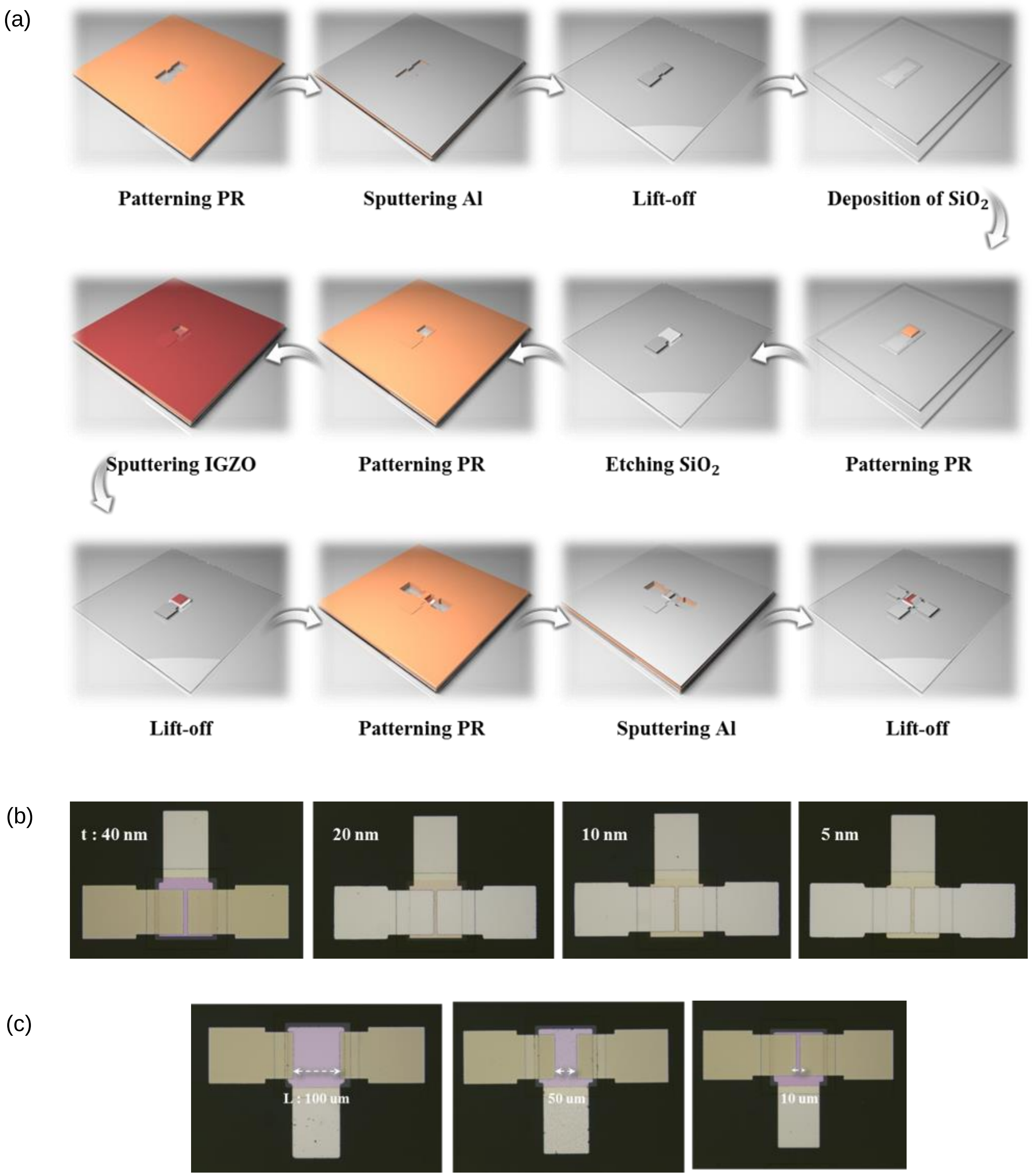

Supplementary Figure S3 | Fabrication of α-IGZO TFTs. Fig S4 (a) shows the fabrication process of α-IGZO TFT. A glass was used as a substrate, the photoresist (PR) (AZ GXR-601) was spin-coated and patterned by using photolithography process. The gate electrode with 150 nm thick Al was deposited by using radio frequency (RF) magnetron sputtering system and lifted off in acetone and isopropyl alcohol (IPA) at ultrasonic condition. The 200 nm thick SiO_2 gate insulator was deposited by plasma enhanced chemical vapor deposition (PECVD) and etched by using reactive ion etching system with CF_4 gas and CHF_3 gas. After that, the active layer of the 80 nm thick α-IGZO ($InGaZnO_4$ target) semiconductor was sputtered by the RF magnetron sputtering system at a gas mixing ratio of Ar/ O_2 = 50/5 (sccm/sccm), and at input power of 400 W and pressure of 5 mTorr, respectively. Then, the α-IGZO was wet-etched by using buffered oxide etchant (BOE) 6:1 and annealed at 300 ˚C for 1 hour in ambient oxygen. Finally, the source-drain electrodes were patterned with 150 nm thick Al. The fabricated α-IGZO TFTs with various t_c and L_c are represented in Fig S4 (b-c) respectively.

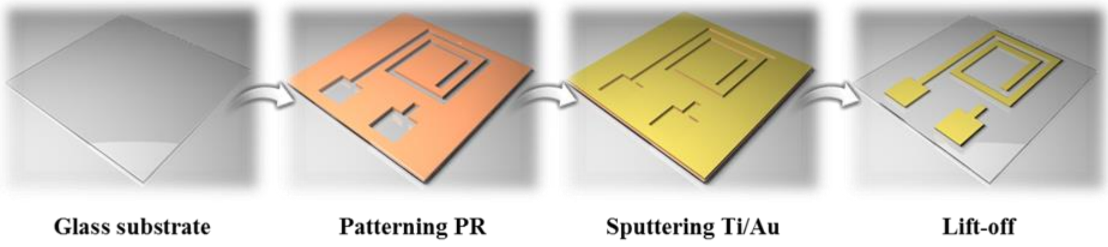

Supplementary Figure S4 | Fabrication process of Coil A. After pattering the PR with thickness of 2 μm, Ti and Au were deposited with about 50 nm and 500 nm thickness respectively onto glass substrate by using RF magnetron sputtering system at an input power of 200 W and the pressure of 5 mTorr. Then, the micro coil was obtained after lift-off process.

(a)

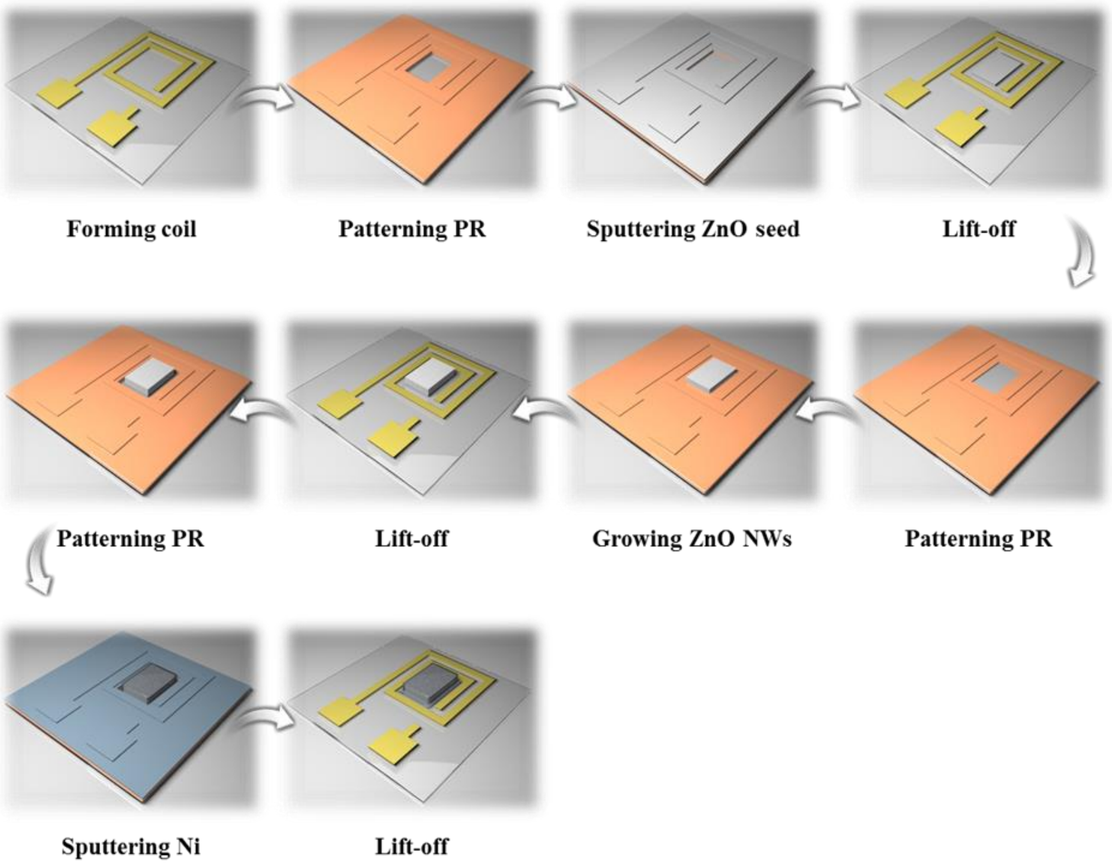

(b)

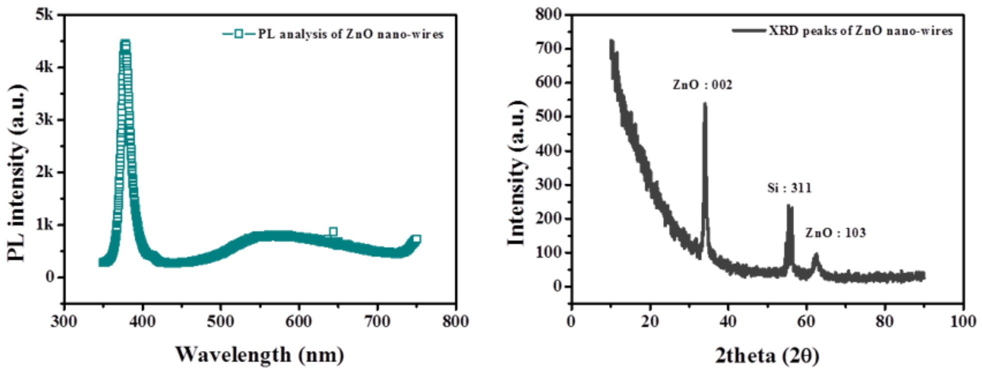

Supplementary Figure S5 | Fabrication process of Coil C and properties of ZnO NWs. Fig S6 (a) gives the fabrication process of Coil C structures. After forming the micro coil, the 50 nm ZnO seed layer was patterned by using the RF magnetron sputtering system and a photolithography process in the center of coil loop. After lift-off process of the ZnO seed layer, we used the hydrothermal process which is possible to grow uniformly ZnO NWs on the substrate. The sample was suspended upside down direction using plastic tools and was then dipped in a glass bottle filled with 20 mM solution which was mixed by hexa-methylenetetramine and zinc nitrate hexahydrate. The nutrient solution was composed of a 1:1 ration of zinc nitrate hexahydrate and hexamethylenetetramine (HMTA). Zinc nitrate salt provides Zn^{2+} ions required for building up ZnO NWs. Water molecules in the solution provide O_2^- ions. The glass bottle was heated in a vacuum oven at 70 ˚C for 48 hours. The grown ZnO NWs were rinsed with an IPA solution to clean the surface of the sample. Then the Ni layer patterned on the vertical ZnO NWs. Fig S6 (b) shows the measurement of X-ray diffraction (XRD) and photo-luminescence (PL) spectrum are the preferred orientation of ZnO NWs. The measurement shows that ZnO NWs are formed as a single crystal.

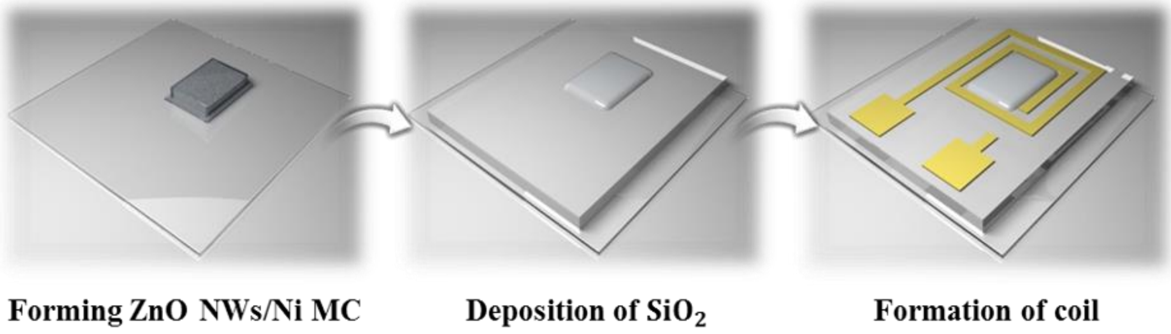

Supplementary Figure S6 | Fabrication process of Coil E. After forming the ZnO NWs/Ni MC structure, SiO_2 was deposited by PECVD system. Then, the micro coil was patterned as aligned with preformed MC structure.

(a)

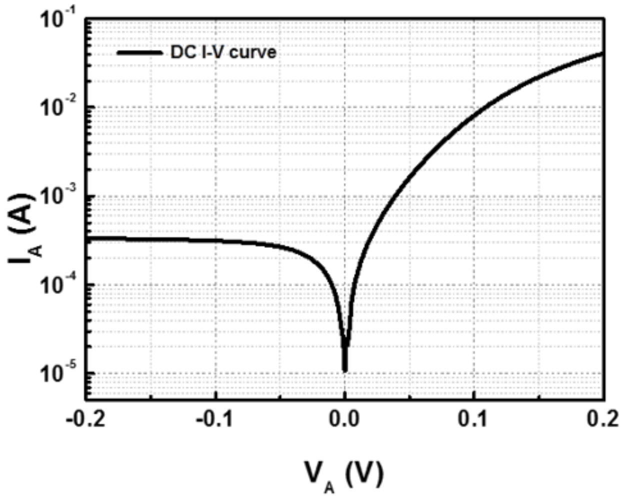

(b)

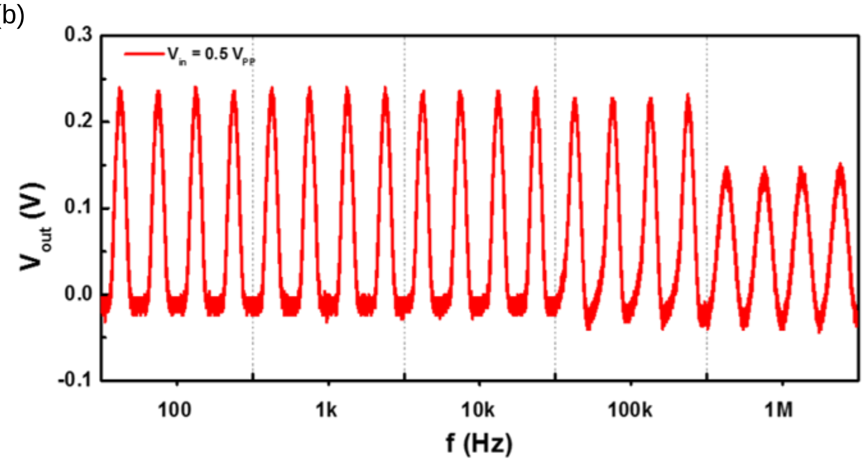

Supplementary Figure S7 | Electrical characteristics of Schottky diode. Figure S7 (a) shows the DC current-voltage characteristic curve of Schottky diode. When forward bias is applied, high anode current (I_A) flows through the diode at a small anode voltage (V_A). The lower voltage drop can serve higher switching speed and better system efficiency. When the diode is reversely biased, relatively lower reverse leakage current passes through the diode. Figure S7 (b) shows the frequency response of the Schottky diode. The Schottky diode can be operated up to high frequency level, which the high cutoff frequency is observed over 100 kHz.
